# Cloning, expression and purification of the α-carbonic anhydrase from the mantle of the Mediterranean mussel, *Mytilus galloprovincialis*

**DOI:** 10.1080/14756366.2017.1353502

**Published:** 2017-07-25

**Authors:** Rosa Perfetto, Sonia Del Prete, Daniela Vullo, Vincenzo Carginale, Giovanni Sansone, Carmela M. A. Barone, Mosè Rossi, Fatmah A. S. Alasmary, Sameh M. Osman, Zeid AlOthman, Claudiu T. Supuran, Clemente Capasso

**Affiliations:** a Istituto di Bioscienze e Biorisorse, CNR, Napoli, Italy;; b Laboratorio di Chimica Bioinorganica, Polo Scientifico, Università degli Studi di Firenze, Sesto Fiorentino, Florence, Italy;; c Dipartimento di Biologia, Università degli Studi di Napoli, Federico II, Napoli, Italy;; d Dipartimento di Agraria, Università degli Studi di Napoli, Federico II, Portici, Napoli, Italy;; e Dipartimento Neurofarba, Sezione di Scienze Farmaceutiche, Università degli Studi di Firenze, Sesto Fiorentino, Florence, Italy;; f Department of Chemistry, College of Science, King Saud University, Riyadh, Saudi Arabia

**Keywords:** Carbonic anhydrase, metalloenzymes, α-class enzyme, hydratase activity, mussel, multidomain protein, protonography, bivalve

## Abstract

We cloned, expressed, purified, and determined the kinetic constants of the recombinant α-carbonic anhydrase (rec-MgaCA) identified in the mantle tissue of the bivalve Mediterranean mussel*, Mytilus galloprovincialis*. In metazoans, the α-CA family is largely represented and plays a pivotal role in the deposition of calcium carbonate biominerals. Our results demonstrated that rec-MgaCA was a monomer with an apparent molecular weight of about 32 kDa. Moreover, the determined kinetic parameters for the CO_2_ hydration reaction were *k*_cat_* = * 4.2 × 10^5^ s^−1^ and *k*_cat_/*K*_m_ of 3.5 × 10^7^ M^−1^ ×s^−1^. Curiously, the rec-MgaCA showed a very similar kinetic and acetazolamide inhibition features when compared to those of the native enzyme (MgaCA), which has a molecular weight of 50 kDa. Analysing the SDS-PAGE, the protonography, and the kinetic analysis performed on the native and recombinant enzyme, we hypothesised that probably the native MgaCA is a multidomain protein with a single CA domain at the *N*-terminus of the protein. This hypothesis is corroborated by the existence in mollusks of multidomain proteins with a hydratase activity. Among these proteins, nacrein is an example of α-CA multidomain proteins characterised by a single CA domain at the *N-*terminus part of the entire protein.

## Introduction

Carbonic anhydrases superfamily (CAs, EC 4.2.1.1) are metalloenzymes, which have been found in all the three domains of life (Eubacteria, Archaea, and Eukarya) and represent a very interesting example of convergent/divergent evolution phenomenon with seven known families: α-, β-, γ-, δ-, ζ-, η-, and θ-CAs^1–3^. In fact, despite the low sequence similarity existing between the seven known CA families, they evolved analogous structures characterised by the following features: (i) catalyse a simple but physiologically relevant reaction consisting in the hydration of carbon dioxide to bicarbonate and protons^4–8^; (ii) the catalytically active form of the enzyme is the metal hydroxide derivative^1–3^; (iii) the rate determining step of the entire catalytic turnover is the formation of the metal hydroxide species of the enzyme by the transfer of a proton from the metal-coordinated water molecule to the surrounding solvent^2–4^^,^^6–17^. The CA macromolecules are grouped in the seven different classes mainly on the basis of their structural fold and arrangement of the active site residues. The α-, β-, δ-, η-, and perhaps θ-CAs are characterised by a Zn(II) ion in the active site. γ-CAs are probably Fe(II) enzymes, although this family is also active with bound Zn(II) or Co(II) ions^18–25^. ζ-CAs are cambialistic enzymes, active both with Cd(II) or Zn(II) bound within the active site^26–28^. The metal ion from the CA active site is coordinated by three His residues in the α-, γ-, δ- and, probably, θ-classes; by one His, and two Cys residues in β- and ζ-CAs or by two His and one Gln residues in the η-class, with the fourth ligand being a water molecule/hydroxide ion acting as nucleophile in the catalytic cycle of the enzyme^1^^,^^5–7^^,^^29^^,^^30^. Some of the catalytically active α-CAs also catalyse the hydrolysis of esters/thioesters, e.g. 4-nitrophenyl acetate (4-NpA) hydrolysis, as well as other hydrolytic reactions. However, no esterase activity was detected so far for enzymes belonging to the other five CA genetic families. The tri-dimensional fold of the five CA classes is very different: α-CAs are normally monomers and rarely dimers; β-CAs are dimers, tetramers, or octamers; γ-CAs are trimers^19^^,^^20^^,^^23^^,^^31^. The only ζ-CA crystallised so far has three slightly different active sites on the same polypeptide chain, whereas no X-ray crystal structures of δ-, η-, and θ-CAs are available so far. All CAs identified in animal systems belong to α-class^32^^,^^33^. CAs identified in plants and algae belong to the α-, β-, γ-, δ-, and θ-classes; fungi encode for α- and β-CAs; protozoa encode for α-, β-, or η-CAs; bacteria encode for enzymes belonging to the α-, β-, and γ-CA classes^4–7^^,^^12^^,^^34^^,^^35^. In metazoans, the α-CA family is largely represented. As described in the literature, CAs play a pivotal role in the deposition of calcium carbonate biominerals in at least 30 metazoan calcifying species^36–41^. In fact, during calcium carbonate formation, the metazoan CAs are involved in the process of acid–base regulation, calcification and mineralisation^39^^,^^41^ providing inorganic carbon at the site of calcification^41^ and/or determining the precipitation of calcium carbonate^42–44^. Recently, we characterised and determined the kinetic constants of the CA purified from the mantle tissue of the bivalve Mediterranean mussel, *Mytilus galloprovincialis*. The protein was indicated with the acronym MgaCA and has been assigned to the α-class of the CA superfamily with the following kinetic parameters for the CO_2_ hydration reaction: *k*_cat_* = *4.1 × 10^5^ s^−1^ and *k*_cat_/*K*_m_ of 3.6 × 10^7^ M^−1^ × s^−147^. The enzyme activity was poorly inhibited by the sulfonamide acetazolamide, with a *K*_I_ of 380 nM. Intriguingly, MgaCA had a molecular weight of 50 kDa, which is roughly two times higher than that of a typical monomeric α-class enzyme (25 kDa)^45^. Here, using the recombinant DNA technology, we prepared and heterologously expressed the recombinant CA (in the text indicated as rec-MgaCA) starting from *N*-amino terminal sequence of the native MgaCA. The catalytic properties of the rec-MgaCA were compared with those obtained for the native enzyme. Our results demonstrated that the rec-MgaCA was a monomer with an apparent molecular weight of 32 kDa and the following kinetic parameters for the CO_2_ hydration reaction: *k*_cat_ = 4.2 × 10^5^ s^−1^ and *k*_cat_/*K*_m_ of 3.5 × 10^7^ M^−1^ × s^−1^. From the comparison of the SDS-PAGE, the protonography, and the kinetic analysis performed on the native and recombinant enzyme, we hypothesised that probably the native MgaCA is a multidomain protein containing a single CA domain, which allows the carbon dioxide hydration reaction.

## Materials and methods

### Gene identification

The rec-MgaCA gene of *M. galloprovincialis* (accession number: ALF62133.1) was identified running the protein “BLAST” program and using the amino acid sequence “SWGYGNDNGP” as query sequence, which is the *N*-amino terminal sequence of the native MgaCA previously determined by the Edman degradation performed on the blotted enzyme^45^.

### Construct preparation, protein expression and purification

The GeneArt Company, specialized in gene synthesis, designed the synthetic *M. galloprovincialis* gene encoding for the α-CA, and containing four base pair sequences (CACC) necessary for directional cloning at the 5′ end of the rec-MgaCA gene. The fragment was subsequently cloned into the expression vector pET100/D-TOPO (Invitrogen, Waltham, MA), creating the plasmid pET100D-Topo/rec-MgaCA. In order to confirm the integrity of the *M. galloprovincialis* gene and the fact that no errors occurred at the ligation sites, the vector containing the fragment was sequenced. *Escherichia coli* ArcticExpress (DE3)RIL competent cells were transformed with pET100/D-Topo/rec-MgaCA, grown at 37 °C, induced with 1 mM IPTG. Zn(SO_4_) was added after 30 min and after additional growth for 16 h, cells were harvested and disrupted by sonication at 4 °C in 20 mM buffer phosphate, pH 8.0. Following sonication, the sample was centrifuged at 1200*g* at 4 °C for 30 min. The supernatant was dialysed against 0.02 M phosphate buffer (pH 8.0) containing 0.01 M imidazole at 4 °C and loaded onto a His-select HF Nickel affinity column (1.0 by 1.0 cm, GE Healthcare). The column was equilibrated with 0.02 M phosphate buffer (pH 8.0) containing 0.01 M imidazole and 0.5 M KCl at a flow rate of 1.0 ml/min. The rec-MgaCA elution was performed with 0.02 M phosphate buffer (pH 8.0) containing 0.5 M KCl and 0.3 M imidazole at a flow rate of 1.0 ml/min. Active fractions (1 ml) were collected and combined for a total volume of 5 ml. Subsequently, they were dialysed, concentrated, and analysed by SDS-PAGE. At this stage of purification, the enzyme was at least 95% pure and the obtained recovery was of 1.0 mg of the rec-MgaCA.

### Sequence analysis

Multialignment of amino acid sequences was performed using the program MUSCLE (MUltiple Sequence Comparison by Log-Expectation), a new computer program for creating multiple alignments of protein sequence^46^.

### SDS-PAGE

Sodium dodecyl sulfate SDS-polyacrylamide gel electrophoresis (SDS-PAGE) was performed as described by Laemmli using 12% gels^47^.

### Protonography

Wells of 12% SDS-PAGE gel were loaded with bCA and rec-MgaCA mixed with loading buffer without 2-mercaptoethanol and without boiling the samples, in order to avoid protein denaturation. The gel was run at 150 V until the dye front ran off the gel. Following the electrophoresis, the 12% SDS-PAGE gel was subject to protonography to detect the bCA and rec-MgaCA hydratase activity on the gel as described by Capasso and coworkers^46^^,^^48^^,^^49^.

### Enzyme kinetics

An Applied Photophysics (United Kingdom) stopped-flow instrument has been used for assaying the CA-catalysed CO_2_ hydration activity^50^. Bromothymol blue (at a concentration of 0.2 mM) has been used as indicator, working at the absorbance maximum of 557 nm, with 10 – 20 mM TRIS (pH 8.3) as buffer, and 20 mM Na_2_SO_4_ for maintaining constant the ionic strength (this anion is not inhibitory and has a *K*_I _> 200 mM against this enzyme), following the initial rates of the CA-catalysed CO_2_ hydration reaction for a period of 10–100 s. The CO_2_ concentrations ranged from 1.7 to 17 mM for the determination of the kinetic parameters and inhibition constants. For each measurement at least six traces of the initial 5–10% of the reaction have been used for determining the initial velocity. The uncatalysssed rates were determined in the same manner and subtracted from the total observed rates. Stock solutions of inhibitor (1–10 mM) were prepared in distilled-deionized water and dilutions up to 0.01 μM were done thereafter with distilled-deionised water. Inhibitor and enzyme solutions were pre-incubated together for 15 min at room temperature prior to assay, in order to allow for the formation of the E-I complex. The inhibition constants were obtained by non-linear least-squares methods using the Cheng–Prusoff equation whereas the kinetic parameters for the uninhibited enzymes from the Lineweaver–Burk plots, as reported earlier, and represent the mean from at least three different determinations.

## Results and discussion

### Identification of the full amino acid sequence of the M. galloprovincialis α-CA

**Figure 1.**
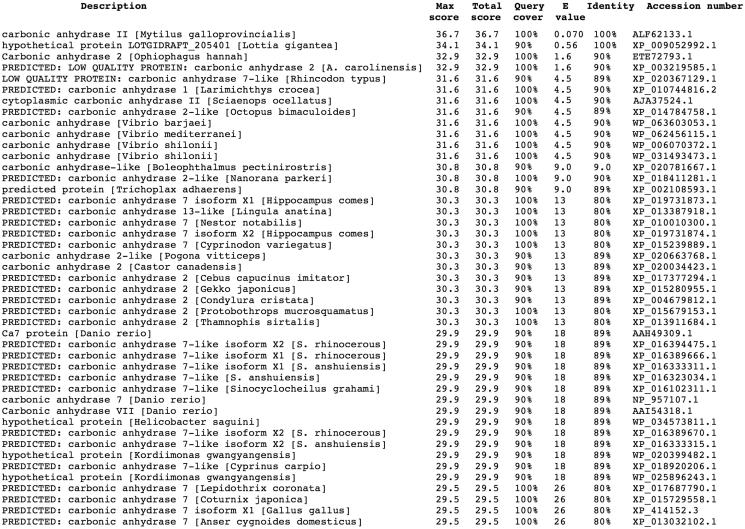
Blast output reporting the CA library sequences. By going down the list, it is possible to see less than perfect matches, slowly degrading as the corresponding score decreases and the E-value increases. The E-value is an assessment of the statistical significance of the score. E-value close to 1 are a warning that the alignment is not reliable.

**Figure 2.**
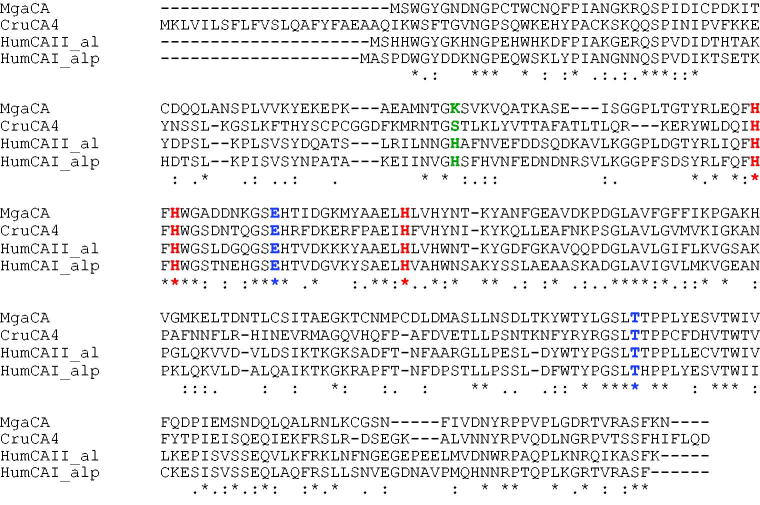
Alignment of α-CA sequences from *Mytilus galloprovincialis* (MgaCA, Accession number: ALF62133.1) *Corallium rubrum* (CruCA4, Accession number: KU557746), *Homo sapiens* I (hCAI, Accession number: NP001729) and II (hCAII, Accession number: P00918), hCA I numbering system was used. The zinc ligands (His94, 96 and 119) and the gate-keeper residues (Glu106 and Thr199) are conserved in aligned sequences; while the proton shuttle residue (His64) is preserved only in the human enzymes. The asterisk (*) indicates identity at all aligned positions; the symbol (:) relates to conserved substitutions, while (.) means that semi-conserved substitutions are observed. Multialignment was performed with the program Muscle, version 3.1.

Using the Basic Local Alignment Search Tool (BLAST) and as query sequence the amino acid sequence “SWGYGNDNGP” deduced by the Edman degradation carried out on the blotted native MgaCA^45^, the full amino acid sequence of the *M. galloprovincialis* α-CA deposited in the NCBI (National Center for Biotechnology Information, USA) library of the protein/enzyme sequences has been identified. The result of BLAST analysis showed that the first amino acid sequence of the top library sequences was the carbonic anhydrases II from *M. galloprovincialis* with a “Query score” and “Identity” of 100% (Figure 1). As shown in Figure 2, the full nucleotide sequence encoding for the native MgaCA showed an open reading frame of 255 amino acid residues containing the conserved three histidines, His94, His96, and His119 (hCA I numbering system), which coordinate the Zn(II) ion crucial for catalysis; and the two gate-keeper residues, the Glu106 and Thr199. The mussel enzyme had a residue of lysine as substituent of the proton shuttle residue His64 (Figure 2), characteristic of the human isozymes, explaining the relatively low catalytic activity of the native MgaCA, with the following kinetic parameters for the CO_2_ hydration reaction: *k*_cat_ = 4.1 × 10^5^ s^−1^ and *k*_cat_/*K*_m_ of 3.6 × 10^7^ M^−1^ × s^−1^ ^45^, with respect to hCA II, which is considered as one of the most active among the α-CAs and the other CA-classes^51^.

### Production of the recombinant enzyme (rec-MgaCA)

**Figure 3:**
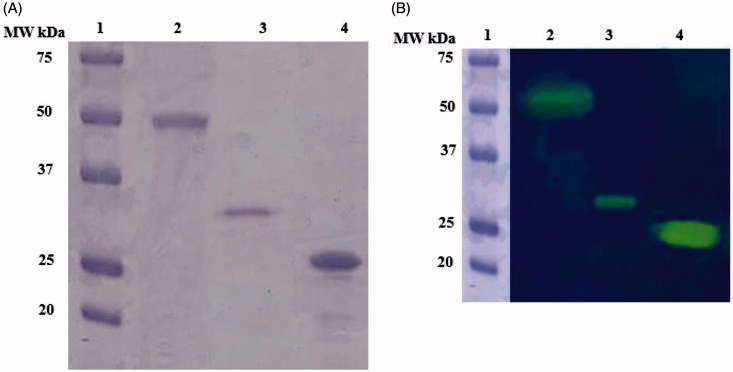
(A and B). A. SDS-PAGE; B. Protonography. In both panels: Lane 1, Molecular weight markers; lane 2, the native MgaCA purified from the mantles of *M. galloprovincialis*; lane 3, the recombinant rec-MgaCA, lane 4, the commercial bovine CA.

The recombinant rec-MgaCA was prepared designing a synthetic gene as described in the section ‘‘Materials and methods’’ and heterologously expressed as a His-Tag fusion protein using the method reported earlier for several CAs^52^. The recombinant enzyme was recovered in the soluble fraction of the *E. coli* ArcticExpress (DE3)RIL cells extract obtained after sonication and centrifugation. Using an affinity column (His-select HF Nickel Affinity Gel), rec-MgaCA was purified to apparent homogeneity, as indicated by SDS-PAGE and protonography (Figure 3(A) and (B), lane 3). The total amount of metalloenzyme recovered was 1 mg. The rec-MgaCA showed a band of about 32 kDa (monomeric form) under reducing condition (Figure 3(A) and (B), lane 3). Intriguingly, the native MgaCA showed a molecular weight of about 50 kDa (Figure 3(A) and (B), lane 2), while the commercial bovine CA (α-CA) had a molecular weight of about 26 kDa (Figure 3(A) and (B), lane 4). Considering the fact that the molecular weight of the rec-MgaCA without the His-Tag is about 30 kDa, its dimer should have a molecular weight of about 60 kDa. As shown in Figure 3(A) and (B), lane 2, it is readily apparent that the native enzyme, MgaCA, showed a molecular weight of approximately 50 kDa, which is 10 kDa lower than that proposed for the dimer (60 kDa). From this analysis, we propose that probably the native enzyme is a multidomain protein characterised by a CA domain present at its *N*-amino terminal sequence and another domain of about 20 kDa at the *C*-terminus. Our hypothesis is corroborated by the existence of particular α-CAs in mollusks, called nacreins. Nacrein has been identified for the first time in the Japanese pearl oyster *Pinctada fucata*^53^. This protein showed a M.W. of 50 kDa and is involved in the nacreous layer formation of shell and pearl. It possesses a hydratase activity because it has a CA domain at the *N*-terminus part of the entire molecule.

### Kinetic analysis comparison

The kinetic parameters for the CO_2_ hydration catalysed by the rec-MgaCA were measured and compared to the human isoforms hCA I, hCA II^54^ as well as to the previous CAs cloned in *Stylophora pistillata*, STPCA (SpiCA1)^43^^,^^55^ and STPCA2 (SpiCA2); CruCA4 from *Corallium rubrum*^40^^,^^43^ and the native CA purified from the mantle of *M. galloprovincialis* (MgaCA) (Table 1)^45^. The kinetic constants of the MgaCA and rec-MgaCA are two times higher than the hCA I enzyme. This is very intriguing since mussel α-CA respect to the hCA I lacked a His64, which is involved in the transfer of a proton from the water coordinated to the Zn(II) ion to the environment with the function to accelerate the rate of the catalytic cycle. Curiously, the recombinant enzyme (rec-MgaCA) has a very similar kinetic and acetazolamide inhibition features, which are comparable with those of the enzyme isolated from the living mussels (within the limits of the experimental error). Thus, it is possible that in the conditions of the assay the recombinant enzyme dimerizes or, as we described in the previously paragraph, the MgaCA isolated from the mussels could have a CA domain and another domain, which is not connected to the catalytic function, and as thus, should not be a dimer but a multidomain protein. Of course, work is in progress in our laboratories to verify the multidomain nature of the native MgaCA.

**Table 1.** Kinetic parameters for the CO_2_ hydration reaction catalysed by the rec-MgaCA, the purified native mussel CA (MgaCA), the *Homo sapiens* CA isoforms (hCA I and hCA II) and coral CA isoforms (SpiCA1 and SpiCA2 from *Stylophora pistillata*; CruCA4 from *Corallium rubrum*). Acetazolamide (AAZ) inhibition data are also shown.

Enzyme	Class	*k*_cat_ (s^−1^)	*k*_cat_/*K*_M_ (M^−1^ × s^−1^)	*K*_I_ (acetazolamide) (nM)
hCA I	α	2.0 × 10^5^	5.0 × 10^7^	250
hCA II	α	1.4 × 10^6^	1.5 × 10^8^	12
SpiCA1	α	3.1 × 10^5^	4.6 × 10^7^	16
SpiCA2	α	5.6 × 10^5^	8.3 × 10^7^	74
CruCA4	α	2.4 × 10^5^	5.2 × 10^7^	450
MgaCA	α	4.1 × 10^5^	3.6 × 10^7^	380
rec-MgaCA	α	4.2 × 10^5^	3.5 × 10^7^	361

Errors in the range of ±5% of the reported data from three different assays.

## Conclusions

As described in the literature, mollusks contain multidomain proteins with hydratase activity^53^^,^^56^. For example, the nacrein is physiologically involved in the nacreous layer formation of shell and pearl^53^. It showed a MW of 50 kDa and has been identified for the first time in the Japanese pearl oyster *Pinctada fucata*. Nacrein is able to convert the carbon dioxide to bicarbonate and protons because it has a single CA domain at the *N*-terminus part of the entire protein. Successively, two novel nacrein-like proteins with CA catalytic function and playing a key role in shell biomineralisation were identified from the shell-forming mantle of the Pacific oyster, *Crassostrea gigas*^57^. Again, the CA encoded by the genome of *Tridacna gigas* represents an example of a α-CA multidomain protein with two CA domains. In fact, this α-CA is a glycoprotein, which has MW of 70 kDa and contains two complete carbonic anhydrase domains within the protein, one at the *N*-terminus, the other at the *C*-carboxy-terminal parts of the protein^56^. The dual domain structure could have arisen from the fusion of two separate CA genes or by a duplication of a single gene followed by a fusion event^58^. Interesting to note that the dual domain CAs have also been previously reported for two algal species, *Dunaliella salina* and *Porphyridium purpureum*^59^^,^^60^. These observations and the results obtained from the SDS-PAGE, protonography, and kinetic analysis give strength to our hypothesis that probably the native MgaCA is a multidomain protein with a single CA domain at the *N*-terminus of the protein. Moreover, the heterologous expression in *E. coli* of the recombinant protein resulted in a valid method for producing a discrete amount of the active rec-MgaCA. This will make possible the use of the biocatalyst either free or immobilised in the CO_2_ biomimetic capture process.
